# HDAC Up-Regulation in Early Colon Field Carcinogenesis Is Involved in Cell Tumorigenicity through Regulation of Chromatin Structure

**DOI:** 10.1371/journal.pone.0064600

**Published:** 2013-05-28

**Authors:** Yolanda Stypula-Cyrus, Dhwanil Damania, Dhananjay P. Kunte, Mart Dela Cruz, Hariharan Subramanian, Hemant K. Roy, Vadim Backman

**Affiliations:** 1 Biomedical Engineering Department, Northwestern University, Evanston, Illinois, United States of America; 2 Department of Medicine, NorthShore University HealthSystem, Evanston, Illinois, United States of America; 3 Department of Medicine, Boston Medical Center, Boston, Massachusetts, United States of America; Goethe University, Germany

## Abstract

Normal cell function is dependent on the proper maintenance of chromatin structure. Regulation of chromatin structure is controlled by histone modifications that directly influence chromatin architecture and genome function. Specifically, the histone deacetylase (HDAC) family of proteins modulate chromatin compaction and are commonly dysregulated in many tumors, including colorectal cancer (CRC). However, the role of HDAC proteins in early colorectal carcinogenesis has not been previously reported. We found HDAC1, HDAC2, HDAC3, HDAC5, and HDAC7 all to be up-regulated in the field of human CRC. Furthermore, we observed that HDAC2 up-regulation is one of the earliest events in CRC carcinogenesis and observed this in human field carcinogenesis, the azoxymethane-treated rat model, and in more aggressive colon cancer cell lines. The universality of HDAC2 up-regulation suggests that HDAC2 up-regulation is a novel and important early event in CRC, which may serve as a biomarker. HDAC inhibitors (HDACIs) interfere with tumorigenic HDAC activity; however, the precise mechanisms involved in this process remain to be elucidated. We confirmed that HDAC inhibition by valproic acid (VPA) targeted the more aggressive cell line. Using nuclease digestion assays and transmission electron microscopy imaging, we observed that VPA treatment induced greater changes in chromatin structure in the more aggressive cell line. Furthermore, we used the novel imaging technique partial wave spectroscopy (PWS) to quantify nanoscale alterations in chromatin. We noted that the PWS results are consistent with the biological assays, indicating a greater effect of VPA treatment in the more aggressive cell type. Together, these results demonstrate the importance of HDAC activity in early carcinogenic events and the unique role of higher-order chromatin structure in determining cell tumorigenicity.

## Introduction

Higher-order chromatin structure regulates a number of biological processes on different scales of organization. Chromatin modulation is well studied at the nucleosome level, which consists of DNA wrapped tightly around a histone octamer composed of the four core histone proteins (H3, H4, H2A, H2B). The core histones are subject to a variety of post-translational modifications on their N-terminal tails, such as methylation, phosphorylation, and acetylation [1]. Given that chromatin structure plays a significant role in gene transcription, dysregulation of proper chromatin structure is present in many diseases. Changes in chromatin structure orchestrate the alterations in tumor suppressor genes or activation of proto-oncogenes needed for neoplastic progression. Thus, epigenetic regulation of gene expression is emerging as an important facet of carcinogenesis, including colorectal cancer (CRC) [2].

Progression of CRC is coordinated by a series of mutations and chromosomal deletions of key oncogenes (e.g., Kras) or tumor suppressor genes (e.g., p53, APC), or through defects in DNA mismatch repair genes (e.g., hMLH1, hMSH2) [3], [4], [5]. In recent years, more attention has been drawn to the interplay of mutational and epigenetic events in both the initiation and progression of CRC [6], [7]. Many of these genetic/epigenetic events are observed distant from where the actual tumor develops, including global methylation [8], [9]. These studies support the concept of field carcinogenesis (also known as field cancerization, field effect, and field defect), which is the proposition that the genetic/environmental milieu that results in a focal neoplastic lesion is present throughout the affected organ [10]. Thus, the genetic and environmental alterations in the diffuse field provide a background on which individual tumors and lesions arise [11]. Our group has recently demonstrated that there are profound nano-scale chromatin alterations in the uninvolved mucosa that may serve as a diagnostic marker of field carcinogenesis [12], [13], [14]. Given the significant and diverse consequences following modifications of chromatin structure and function, it is crucial to identify the underlying physical and biological mechanisms during early stages of field carcinogenesis.

There are several proteins that play a role in determining higher-order chromatin architecture. One group of mediators in chromatin structure is the histone deacetylase (HDAC) family of proteins. The HDAC proteins have important biological function in removing the acetyl group from histones, which in turn promotes compaction of chromatin and influences transcription [15], [16]. In addition to deacetylation of core histones, HDACs can also affect protein stability, protein-protein interactions, DNA binding, and protein localization [16]. These crucial biological functions of HDACs have important implications in the pathogenesis of many diseases, especially cancer. Indeed, there are reports supporting significant up-regulation of HDACs in breast, colorectal, and prostate tumors [17], [18], [19], [20]. Despite significant evidence that chromatin modifications play a major role in cancer development, there is limited information on HDAC regulation in early and field carcinogenesis.

In this study, we analyzed the expression of several HDACs in the uninvolved mucosa (i.e. “field”) of human colon cancer. We then selected HDAC2 as a candidate biomarker, as it had previously been shown to be up-regulated in colorectal carcinoma and it is localized only in the nucleus [21]. To determine if HDAC2 could potentially serve as an early biomarker of colorectal cancer, we also analyzed HDAC2 expression in the azoxymethane (AOM)-injected rat model that mimics early stages of the adenoma-carcinoma sequence of human CRC. Previous research has indicted that HDAC inhibitors (HDACIs) target more malignant cells, although the mechanism of action remains largely unknown. Therefore, to more precisely study the role of HDACs in cancerous transformation, we also treated genetic variants of HT-29 colon cancer cell lines that model varying aggressiveness with a pharmacological HDAC inhibitor (HDACI). HDACIs are small molecules that interfere with HDAC activity and are currently being tested in clinical trials for cancer therapy. These inhibitors induce a variety of cellular effects by reactivating suppressed genes involved in tumor-cell growth and apoptosis regulation [22], [23], [24]. We selected one such agent, valproic acid (VPA), which inhibits Class I and IIA HDACs and is actively studied for cancer therapeutics. We analyzed the effect of VPA on chromatin nano-architecture using micrococcal nuclease (MNase) digestions, transmission electron microscopy (TEM), and a novel optical imaging method (partial wave spectroscopy, PWS). We found that HDAC inhibition by VPA treatment had a greater effect on nuclear nano-structure and function in the more aggressive cell lines. These results indicated that HDACs are a novel marker of human colon field carcinogenesis and support our hypothesis that HDACIs target more tumorigenic cell types related to changes in nuclear mass density, chromatin accessibility, and cell viability.

## Methods

### Tissue Samples and RNA Isolation

Human colon resections and rectal biopsies were obtained from patients at NorthShore University HealthSystem in Evanston, IL, following the Institutional Review Board (IRB) guidelines. Written consent forms were approved by the NorthShore University HealthSystem IRB. All animal procedures were reviewed and approved by the Institutional Animal Care and Use Committee for NorthShore University HealthSystem. Fisher 344 rats (Harlan, Indianapolis, IN) on a standard AIN76a diet were treated with 2 weekly injections (i.p.) either of 15 mg/kg AOM or saline (Midwest Research Institute, Kansas City, MO). Rats were euthanized after 10 weeks (pre-malignant time point). Total RNA was isolated from tissue using TRIzol reagent (Molecular Research Center, Inc., Cincinnati, OH) according to manufacturer’s instructions.

### Quantitative RT-PCR

Complementary DNA (cDNA) synthesis was performed using a high capacity cDNA synthesis kit (Applied Biosystems by Life Technologies, Carlsbad, CA), following standard protocol. The pre-amplified human cDNA was then diluted with TaqMan Universal Mastermix (Life Technologies, Carlsbad, CA) and loaded on the custom-designed TaqMan Low Density Array (TLDA) card and run on ABI Prism 7900 HT PCR machine. The real time PCR data analysis was done using SDS RQ Manager 1.2. All individual HDAC2 PCR reactions were carried out using 80 nM of the TaqMan probe and PCR Mastermix (Applied Biosystems, Carlsbad by Life Technologies, Carlsbad, CA) in a Cepheid Smart Cycle (Cepheid, Sunnyvale, CA). All samples were normalized to β-actin, and the average fold differences were calculated using the comparative Ct method [25]. Threshold of fold change significance was set as >1.5 (up-regulation) and <0.67 (down-regulation).

### Cell Lines and Cell Proliferation Assay

HT-29 cells were grown in McCoy’s 5A medium (ATCC, Manassas, VA) mixed with 10% fetal bovine serum +50 mg/mL penicillin/streptomycin in a 5% CO_2_ environment at 37°C. C-terminus Src kinase (CSK) shRNA-stably transfected HT-29 cells were selected as a clonal population and grown as previously described [26]. Knockdown of the tumor suppressor CSK results in increased aggressiveness compared to control HT-29 cells, as previously measured by both biological assays and PWS [26], [27], [28], [29]. First, knockdown of CSK in the constructs was confirmed for these experiments using qRT-PCR and Western Blotting methods, compared to controls. Various concentrations of VPA (Sigma, St. Louis, MO) were applied to cells based on previous publications [30]. Additional concentrations of VPA (0.1 mM-2.0 mM) and incubation times (6–48 hours) were tested to minimize cell toxicity in the HT-29 cell lines. Target concentrations and treatment times were then used for subsequent studies. At the end of the incubation with VPA, the media was replaced with fresh media containing WST-1 (4-[3-(4-iodophenyl)-2-(4-nitrophenyl)-2H-5-tetrazolio]-1, 3-benzene disufonate) reagent (Roche Diagnostics, Indianapolis, IN). After 30 min incubation with WST-1, the absorbance of the plate was read at 440 nm and 600 nm in a Spectramax Plus Spectrophotometer plate reader (Molecular Devices, Sunnyvale, CA).

### PWS System and Analysis

PWS is a novel imaging technique that measures the nanoscale (∼20 nm) distribution of mass density. The in-depth explanation of the theory and PWS instrument used for this study has been reported elsewhere [13], [27], [29]. In brief, a spatially incoherent white light illuminates a specimen and the reflected back-scattered image is projected on to a CCD camera (Princeton Instruments, NJ, USA) through a spectral tunable filter. The spectral fluctuations of the backscattered light (500–700 nm) are analyzed for every pixel of the obtained image. The PWS technique breaks up the 3-dimensional intracellular volume of a sample into several 1-dimensional cylindrical channels; the multiple interference of backscattered light waves from each of those channels results in the PWS signal. The underlying physical phenomenon is that the optical interference of backscattered light waves is sensitive to the spatial variations of the optical refractive index at sub-diffractional length scales. For biologically relevant macromolecules (DNA, RNA, proteins, lipids, etc.), the refractive index is a linear function of the local macromolecular density [31], [32]. Thus, PWS can quantify spatial variations of macromolecular density.

The PWS readout is an image (2-dimensional [2-D] map) of a cell showing the intracellular distribution of a parameter called disorder strength (*L_d_*), which captures the spatial fluctuations of macromolecular density. *L_d_* is defined as: 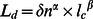 where 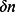 is the standard deviation of the refractive-index (i.e., mass-density) variations, while 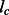 is the correlation length of these variations. The coefficient α depends on the cytology sample preparation (α = 1 in our case), while β depends on the configuration of the optical set up (∼1 for this study). As *L_d_* is a measure of the spatial variations of macromolecular density, it increases with macromolecular condensation [33]. The precise nature of the compaction depends on the intracellular location where *L_d_* is increased. We used the 2-D map, 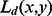, of each nuclear region and calculated the mean nuclear 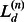 (the average over *x* and *y* pixels). This 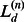 and the standard error calculated from its standard deviation 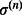 are depicted in all bar plots presented in this manuscript. We randomly selected 50–80 cells for each control and treatment group over multiple experimental repeats to account for any cell variations. The standard deviation of the 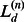 for different sets of slides and repeats was negligible within each group. All *p* values were calculated using Student’s *t* tests using Microsoft Excel.

### MNase Accessibility Assays

Assays were carried out using a modified protocol to previously described methods [34]. Briefly, living cells were washed with PBS, treated with NP-40 lysis buffer, and then incubated with MNase digestion buffer containing 200 Units/ml MNase (Sigma, St. Louis, MO). The digestion was stopped at various time points (0, 2, 5 and 10 minutes) using a 5 M EDTA/10% SDS solution. DNA was purified by phenol/chloroform extraction and ethanol precipitation, following standard methods. To visualize the digestion assay, 2.5 µg of the 0′ MNase samples and 10 µg of the remaining samples were loaded on a 1.2% agarose gel.

### TEM Cell Preparation and Imaging

HT-29 cell lines were treated with target VPA concentrations for 24 hours. After incubation with VPA, cell pellets were collected by centrifugation at 900 rpm. To maintain good structural morphology, both the cell pellets and tissue samples were then immediately high pressure frozen using a Leica EM-PACT2 high-pressure freezer at the Biological Imaging Facility of Northwestern University. Automatic freeze substitution was performed using a Leica AFS2 system. Samples were then embedded in Epon 812 resin (Electron Microscopy Sciences, Hatfield, PA) and thin-sectioned into 90 nm sections onto copper grids using a Leica Ultracut S microtome. High-resolution digital images of samples were collected using a JEOL 1230 and Advanced Microscopy Techniques imaging software at Northwestern University.

## Results

### Chromatin Rearrangement and HDAC2 Up-regulation Occurs Early in the Field of Colorectal Carcinogenesis

**Figure 1:**
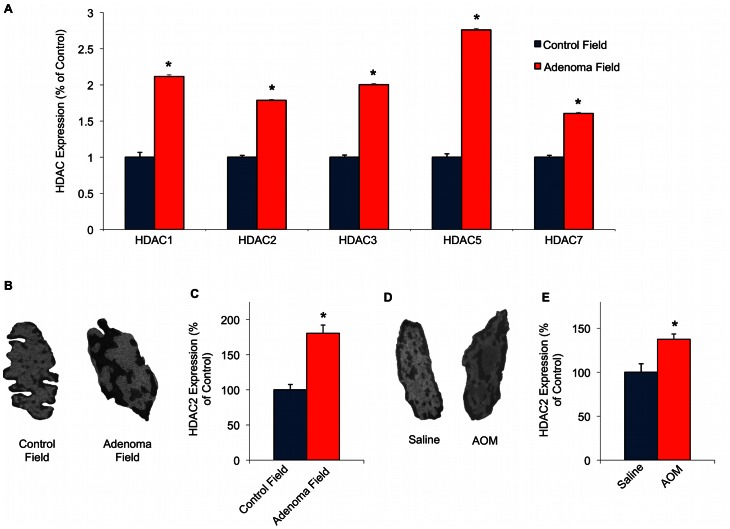
HDAC2 expression is up-regulated in human field carcinogenesis and early carcinogenesis. **A)** mRNA expression of HDAC1, HDAC2, HDAC3, HDAC5, HDAC7 in human field carcinogenesis from (n = 86, patients with adenomas vs. controls). **B)** Representative TEM images of nuclei in histologically normal rectal cells from patients with or without adenomas elsewhere in the colon. **C)** Up-regulation of HDAC2 in field carcinogenesis was confirmed in human resection samples by qRT-PCR methods (n = 12, patients with adenomas vs. controls). **D)** Representative TEM images of saline-injected or azoxymethane-injected (AOM) nuclei obtained from the distal colon at a premalignant time point. **E)** HDAC2 expression is also up-regulated in the AOM (azoxymethane-injected) rat model for early colorectal carcinogenesis (n = 12 animals). Standard error bars shown with *p<0.05.

In colorectal carcinogenesis, many genetic/epigenetic modifications that lead to a focal tumor exist throughout the organ, referred to as field carcinogenesis [8], [9], [10]. In previous studies, our group demonstrated that nanoscale alterations in chromatin structure occur in human field carcinogenesis [12], [13], [14]. To determine molecular mediators of these alterations, we took a candidate approach and considered the HDAC family of proteins. HDAC proteins regulate chromatin structure and genome function and are frequently dysregulated in many cancers [35]. We performed a PCR array for HDAC1, HDAC2, HDAC3, HDAC5 and HDAC7 on human rectal biopsies from patients with or without an adenoma in the colon (n = 86 subjects total). We found that each HDAC was significantly up-regulated (*p*<0.001; Figure 1A). To confirm the nanoscale alterations in chromatin structure, we compared TEM images of nuclei from control patients (no dysplasia) and patients with an adenoma (pre-cancerous lesion). The electron micrographs revealed significant nanoscale differences in structure, including increased chromatin compaction, presence of multiple nucleoli, and alterations in the distribution of chromatin in the human rectal biopsies with an adenoma present, compared to control (Figure 1B). We then confirmed the expression of HDAC2 in human field CRC using an individual qRT-PCR. HDAC2 was selected as a target biomarker due its nuclear localization, while many other HDAC family members translocate from the nucleus ad have exhibited protein interaction in the cytoplasm. Additionally, it was previously shown that HDAC2 is overexpressed in colorectal carcinomas but not in the field of the tumor [21]. In human resection samples, we found that HDAC2 expression was also about 2-fold higher in patients harboring an adenoma elsewhere in the colon compared to the control (no dysplasia) group (*p*<0.05, n = 12 subjects; Figure 1C). These findings suggest that HDAC expression, and especially HDAC2, are important mediators of chromatin alterations observed at a distance from the actual tumor site.

Next, we analyzed the HDAC2-mediated effects on chromatin structure at an early time point (10 weeks post initial injection) in the AOM-treated rat model. At this pre-neoplastic time point in the AOM-treated rat model, the colon begins to exhibit the first detectable, pre-neoplastic lesions (such as aberrant crypt foci) [36], [37]. TEM micrographs again revealed changes in chromatin compaction in the AOM-treated rat distal colons compared to controls (Figure 1D). Even though the colons did not have any polyps or tumors, HDAC2 was significantly up-regulated (1.5 fold, *p*<0.05) in the AOM-injected animals compared to their age-matched controls by qRT-PCR methods (n = 12 animals; Figure 1E). These observations support the concept that chromatin rearrangements, through HDAC dysregulation, may be important for colon cancer development and could serve as a marker of field carcinogenesis.

### The HDAC Inhibitor VPA Differentially Affects Colon Cancer Cell Lines with Varying Tumorigenicity

**Figure 2:**
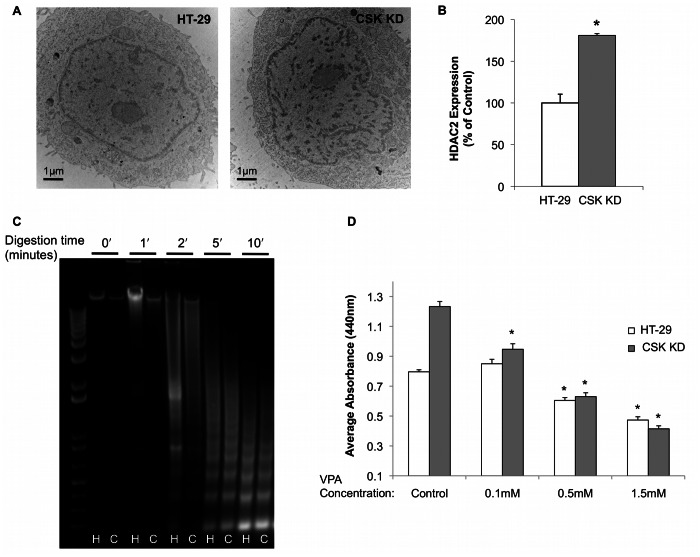
HDAC inhibition differentially affects cell viability in colon cancer cell line variants. **A)** TEM micrographs of chromatin structure in the HT-29 colon cancer cell line genetic variants, HT-29 control and CSK knockdown. **B)** HDAC2 expression is up-regulated in the CSK knockdown cell lines. **C)** MNase assay on HT-29 (H) and CSK knockdown (C) cells also indicate a more compact chromatin structure present in the CSK constructs. **D)** HT-29 and CSK knockdown cells in 96-well plates were treated with increasing concentrations of VPA for 24 h and then assayed for proliferation using standard WST-1 assay. Absorbance was measured after 20 min at 37°C. VPA treatment reduced cell viability in both cell lines, while the effect was greater in the CSK constructs. Standard error bars shown with *p<0.05.

To study the role of HDACs on nuclear nano-structure in cancerous cells, we used human colon cancer cell lines, HT-29 and CSK shRNA-transfected HT-29. Modest knockdown (∼50%) of the tumor suppressor CSK in HT-29 cells results in a more proliferative phenotype; CSK is altered early in the uninvolved colonic mucosa prior to neoplastic transformation, thus serving as a robust cell culture model of early tumorigenic events [26], [29]. In the present study, we observed a 1.5 fold increase in HDAC2 expression in the CSK shRNA knockdown cells compared to HT-29 control cell lines (*p*<0.05; Figure 2A). In agreement with the human and animal data, TEM images showed increased chromatin compaction in the more aggressive (CSK constructs) cell line (Figure 2B).

Chromatin accessibility was evaluated using MNase accessibility assays to compare higher-order chromatin structure in the HT-29 cell lines. HT-29 and CSK shRNA knockdown HT-29 cells were treated with MNase for increasing amounts of time (Figure 2C). In the absence of MNase, the HT-29 cell lines did not exhibit endogenous nuclease activity. The more aggressive CSK shRNA knockdown cells exhibited enhanced resistance to MNase, which indicated a more closed structural state of chromatin [38]. The difference between the cell lines was most apparent at earlier digestion time points.

HDACIs are being studied as a means of inhibiting overexpression of HDACs during carcinogenesis. In order to systematically study the nuclear differences in HT-29 and CSK constructs, we treated the cell lines with an HDAC inhibitor to perturb chromatin architecture. VPA previously has been shown to selectively inhibit Class I and IIA HDACs, as well as HDAC2 expression levels [30], [39]. We first determined the appropriate concentration of VPA and treatment time in HT-29 cell lines using the WST-1 cell viability assay. It has been well established that HDACIs induce apoptosis in cancerous cells [16], [40]; therefore, we targeted the least toxic concentrations on the cells (Figure 2D). Low concentrations of VPA moderately increased cell proliferation in HT-29 cells but not in the CSK shRNA knockdown cells. Similar to previous reports, higher concentrations of VPA treatment (0.5 mM –1.5 mM) reduced cell viability [30]. However, the effect on viability was more pronounced in the CSK shRNA cell lines as compared to the HT-29 cell lines with the same treatment conditions. These results are consistent with the hypothesis that HDAC dysregulation and chromatin rearrangements are involved in cell tumorigenicity.

**Figure 3:**
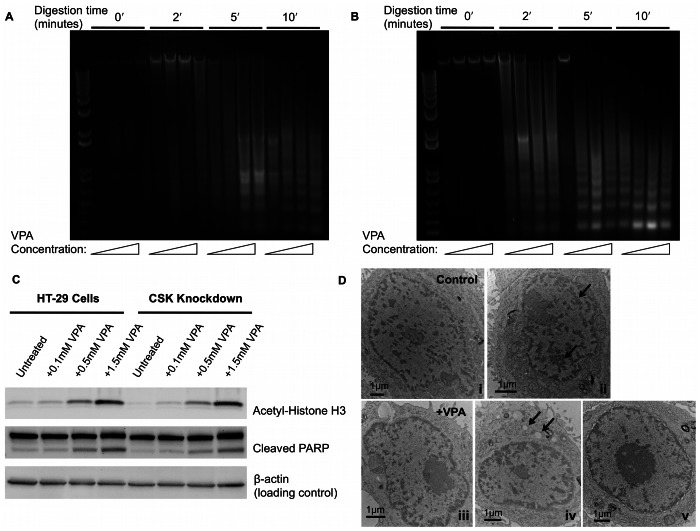
HDAC inhibition increases chromatin accessibility in colon cancer cell line variants. MNase assays of **A)** HT-29 cells and **B)** CSK knockdown cell lines treated with increasing concentrations of VPA for 24 h. **C)** Western blot analysis probing with antibodies against acetyl-histone H3 and apoptotic marker with cleavage of PARP (poly ADP ribose polymerase) in VPA treated HT-29 and CSK knockdown cells. β-actin is shown as a protein loading control. **D)** Representative TEM images showing altered chromatin distribution in the VPA treated samples compared to the control cells.

In addition to cell viability, treatment with HDACIs alters chromatin condensation and accessibility in cancerous cells [41]. To examine the effect on chromatin following VPA treatment in HT-29 and CSK knockdown cells, we performed MNase digestions of isolated nuclei. In agreement with recent publications, we found that HT-29 cells treated with VPA were more sensitive to MNase compared to untreated samples, indicating a more open chromatin structural state (Figure 3A) [41]. At early digestion time points, the shift in DNA bands suggested that there was an increase in accessibility with VPA treatment. However, at later time points, the higher concentrations of VPA (0.5 and 1.5 mM) displayed greater accessibility compared to the untreated sample. On the other hand, digestion of CSK knockdown samples revealed a large effect on DNA accessibility with increasing VPA concentrations (Figure 3B). This indicated that the HDACIs induced greater chromatin changes in the more aggressive cell lines. To verify these higher-order chromatin changes in both cell lines, we performed immunoblotting of acetyl-histone H3. The results showed that there was an increased proportion of acetylation in VPA-treated samples in both HT-29 and CSK constructs (Figure 3C). Because the differences among concentrations in the HT-29 cells may have reflected the apoptotic effects of the HDACIs, we also examined the level of apoptosis induced by VPA. Immunoblotting showed an increase in the cleavage of poly (ADP-ribose) polymerase (PARP), a marker for caspase-mediated apoptosis (Figure 3C). Given that chromatin accessibility and acetyl-histone H3 were assessed on entire cell populations, we next performed TEM imaging to assess nano-scale difference in chromatin between individual cells. TEM imaging confirmed less compact chromatin structures in the VPA-treated nuclei compared to their untreated counterparts (Figure 3D). Furthermore, there was an increase in the number of vacuoles in the cytoplasm among VPA-treated cells, indicating early apoptotic effects of drug treatment. Together, these data show that VPA elicits a greater effect in the more aggressive cell line compared to control, which may relate to the HDAC activity in HT-29 cell lines.

### PWS Quantification of Chromatin Architecture in Colon Cancer Cell Lines

Pharmacological HDACIs target a small subset of genes rather than modulating global gene expression [42]. MNase digestions provide structural information of DNA accessibility on a global level over the entire sample while TEM micrographs show these chromatin alterations qualitatively. Therefore, to quantify local, nano-architectural modifications in individual nuclei, we used the PWS technique. This technique measures nanoscale spatial fluctuations of macromolecular concentration, specifically, macromolecular condensation – via a parameter termed disorder strength (*L_d_*). In the nucleus, *L_d_* is a measure of chromatin compaction, as has been confirmed by numerical simulations [43]. An increase in *L_d_* occurs in the field of several human cancers and at the earliest stages of carcinogenesis in both clinical patients and animal models [13], [29]. Furthermore, previous studies demonstrated that *L_d_* increased for the more aggressive CSK constructs compared to HT-29 control cells [27]. Therefore, the PWS technique was an ideal tool to measure the effect of HDAC inhibition on local chromatin structure and the relationship with cell tumorigenecity. We hypothesized that HDAC inhibition by VPA opens these local chromatin regions, thus normalizing or decreasing the aggressiveness differences between the cell lines as measured by PWS.

**Figure 4:**
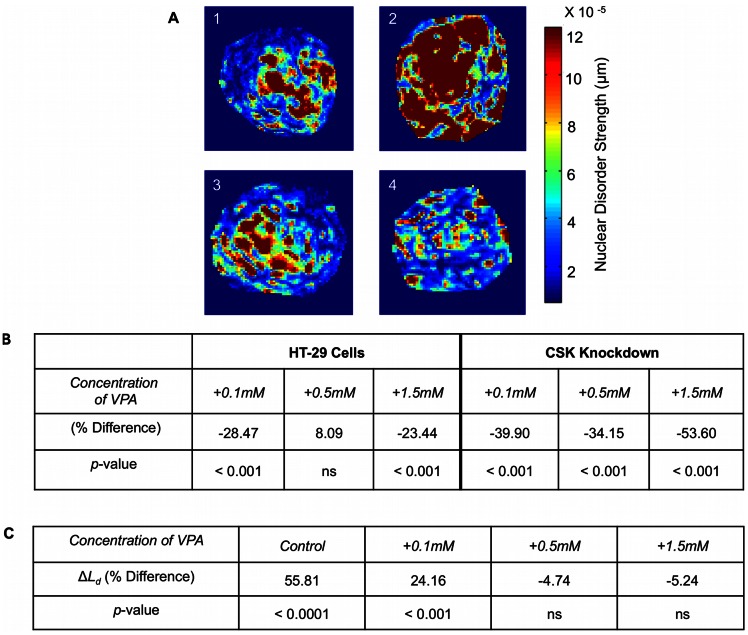
Changes in nuclear disorder strength (L_d_) following VPA treatment. **A)** Representative pseudocolor PWS images from nuclei of HT-29 and CSK constructs untreated or treated with 0.5 mM VPA. Color shows the magnitude of the *L_d_* in an individual nucleus. **B)** Percent difference in combined nuclear *L_d_* over experimental repeats in HT-29 and CSK knockdown cells. Nuclear *L_d_* mostly decreased following VPA treatment in each cell line and to a greater extent in the CSK constructs. **C)** Percent difference in nuclear disorder strength between HT-29 and CSK constructs after each treatment. Treatment with higher concentrations of VPA (0.5 mM and 1.5 mM) nullified the nuclear *L_d_* differences between the cell lines.

We first determined the effect of VPA on HT-29 and CSK constructs individually. Second, we analyzed the disorder strength difference (Δ*L_d_*) between the two cell lines. Upon treatment with VPA, both the HT-29 and CSK knockdown cell lines appeared microscopically similar (Figure 4A). Low concentration of VPA caused *L_d_* to decrease in both the HT-29 cells and CSK knockdown cells (28.47% and 39.9%, respectively; Figure 4B). The effect of 0.1 mM VPA on nuclear *L_d_* was greater in CSK constructs, supporting findings from previous research indicating HDACIs target more malignant cells. In both HT-29 and CSK constructs, treatment with 0.5 mM VPA resulted in a slight increase in *L_d_* compared to the lowest concentration, which may be due to the fact that HDACIs induce apoptosis. However, in the CSK constructs this value remained lower than the untreated samples as opposed to the non-significant increase in the HT-29 cells. Treatment with 1.5 mM VPA caused *L_d_* to further decrease in both cell lines (Figure 4B). The differential effect of HDAC inhibition between cell lines may be attributed to the higher level of HDAC2 expression and higher aggressiveness in the CSK constructs. These results are consistent with the biological assays, showing a greater effect of VPA in the more aggressive CSK constructs.

The loss of CSK is an early carcinogenic event, important for Src signaling and cell hyperproliferation, shown in both cell culture models and tissue samples [26], [28]. To then understand the relationship of CSK loss with nanoscale chromatin rearrangements, we compared the Δ*L_d_* between HT-29 and CSK constructs at specific VPA treatments. The CSK constructs exhibited a higher *L_d_* compared to HT-29 control cell lines (55.81% difference; Figure 4C). Low concentrations of VPA (0.1 mM) decreased *L_d_* significantly in both cell lines, but there was still a 24.16% difference between HT-29 and CSK constructs. On the other hand, higher concentrations of VPA (0.5 mM and 1.5 mM) completely normalized the *L_d_* differences in HT-29 and CSK knockdown cell lines (−4.74% and −5.24% difference, respectively; Figure 4C). These results indicate that increasing concentrations of VPA increased chromatin accessibility, which in turn normalized the Δ*L_d_* between the cancer cell lines. Therefore, in this study, we quantified for the first time the direct relationship between chromatin rearrangements and cellular aggressiveness by comparing nuclear mass-density fluctuations in cancer cell lines. The PWS results support our hypothesis that HDACIs open local chromatin regions, normalizing the aggressiveness between the cell lines.

## Discussion

Proper regulation of higher-order chromatin structures is essential for normal cell function. Abnormal alterations in genome organization leads to aberrant gene expression and has been described in a variety of diseases, including neurological disorders and many cancers [44], [45], [46]. These conditions have prompted increased efforts to understand and determine important histone modifications through histone deacetylase/acetylase activity and mutations during neoplastic development and progression. Microscopically observable changes in nuclear morphology also serve as a marker of carcinogenesis and are also observed in cells within the field of the tumor [12], [47]. Field carcinogenesis is the concept that the genetic/environmental milieu that results in a focal tumor exists not only at that particular location, but affects the organ diffusely. Thus, field carcinogenesis represents the earliest stage of malignancy [11]. However, the cause and mechanisms governing chromatin changes during early stages of field cancerization are largely unknown. For this reason, in this study, we examined how changes in chromatin nano-structure through HDAC regulation are involved in determining the aggressiveness of cells using a multidisciplinary approach. First, we determined the expression of several important HDAC proteins in field colon carcinogenesis using human rectal biopsies. To our knowledge, this is the first study demonstrating that HDAC1, HDAC2, HDAC3, HDAC5, and HDAC7 are up-regulated in the field of human colon cancer. Secondly, we analyzed TEM images of nuclei from these samples to visualize chromatin modifications in the field of CRC. We then validated the expression of a nuclear localized HDAC family member, HDAC2, in colonic resections as a candidate for further study. HDAC2 was significantly increased in the adjacent mucosa obtained from patients harboring an adenomatous polyp (pre-cancerous lesion) compared to non-dysplasia (control) patients using qRT-PCR methods, suggesting that chromatin modifications through HDAC regulation extend throughout the entire field of carcinogenesis. While the up-regulation of HDAC2 has previously been shown by immunohistochemistry in several cancers [19], [21], HDAC2 expression has never been described in early field carcinogenesis.

Increased chromatin compaction and other nuclear abnormalities have long served as a marker of dysplasia. Consistent with these later stage events of carcinogenesis, we found increased chromatin condensation and changes in chromatin distribution in human field carcinogenesis using TEM. Given that these chromatin alterations occur in the diffuse field are known to occur in the tumor, we then assessed the expression of HDAC2 and chromatin nano-structure using TEM in the azoxymethane (AOM)-treated rat model for early CRC. We again observed an increase in chromatin compaction by TEM and found that HDAC2 is also up-regulated at an early, premalignant time point in the AOM-injected rat model, further supporting that concept that chromatin modifications represent early neoplastic events. Together, these results in both the animal model and human patients support the use of HDAC2 as an early biomarker of colon carcinogenesis.

Dysregulation of HDACs causes aberrant gene expression, thus promoting tumor development [23]. For this reason, the HDAC family is being studied as a target for cancer therapy. On a molecular level, HDACIs have been linked to cell cycle regulation and induction of apoptosis through regulation of key cell growth genes like Rb (retinoblastoma) and p21^WAF1^ [48], [49]. Through such pathways, HDACIs selectively target more tumorigenic cells, both *in vitro* and i*n vivo* [15], [35]. Our results of VPA treatment on colon cancer lines also show that increasing concentrations of VPA induced higher apoptosis. Similar to the chromatin changes observed in human and animal tissue, we found increased HDAC2 expression, changes in higher-order chromatin structure, histone acetylation, and altered DNA accessibility in the more aggressive CSK constructs compared to the HT-29 control cells. Moreover, increasing concentrations of VPA treatment reduced cell viability to a greater extent in CSK knockdown cells compared to HT-29 control cells, supporting previous research that indicated HDACIs target more tumorigenic cell types.

To quantify chromatin modifications at the nanoscale level, we used a novel imaging technique (PWS) to assess mass-density fluctuations in individual nuclei. Unlike traditional microscopy, PWS has been shown to successfully differentiate genetically altered human colon cancer cell lines and animal cancer cells that appear microscopically similar [29]. The PWS technique has also demonstrated exquisite sensitivity to detecting early nano-architectural changes in the field of cancerization of lung, pancreas, and colon cancers [13], [14], [29], [50]. On a cellular level, *L_d_* likely correlates to chromatin modifications in the nucleus [51]. Indeed, we found that by reducing chromatin compaction through VPA, nuclear *L_d_* was reduced in both HT-29 and CSK construct cell lines. The decrease in *L_d_* was more significant in the CSK constructs, suggesting that chromatin rearrangements play an important role in the mechanism of HDAC inhibition. The PWS results are consistent with the cell viability assays, MNase digestions, TEM, and immunoblotting of histone acetylation. Treatment with high concentrations of VPA normalized the *L_d_* differences between the two cell lines, thus eliminating their differences in cellular aggressiveness. Taken together, these results support our hypothesis that HDACIs target more cancerous cell types and that rearrangements in higher-order chromatin structure influences the activity of the HDACIs.

These results are relevant to diverse applications: i) HDAC2 as a novel biomarker of the field effect in early colorectal carcinogenesis, ii) the support of HDAC inhibitors and other epigenetic therapies for cancer, and iii) the use of PWS to quantify changes in chromatin architecture of cancer cells. This comprehensive approach of biological assays, microscopy, and spectroscopy demonstrates that nuclear nanoscale alterations are important in the field during early carcinogenesis related to structural mechanisms of biological events. Previous reports demonstrate that *L_d_* is increased in colon, lung, and pancreatic cancers [14], [50]. Therefore, early HDAC dysregulation is likely a universal event in other cancers. Additional research on the mechanisms of HDAC2 and other nanoscale higher-order chromatin modifications will lead to a greater understanding of the earliest stages of carcinogenesis. In this way, PWS can serve as an important tool in addressing questions in cancer biology by assessing chromatin remodeling and other nanoscale structural alterations of chromatin. While we found HDACs to be important in early stages of carcinogenesis, there are a number of other mediators of chromatin alterations that may also play a role (e.g., the SWI/SNF family of proteins). Moreover, future studies on patient samples will verify the ability of PWS to address other clinically relevant epigenetic events, such as methylation and chemoresistance.

## References

[1] Sawan C, Herceg Z (2010) Histone modifications and cancer. Adv Genet 70: 57–85. 20920745 10.1016/B978-0-12-380866-0.60003-4

[2] Lao VV, Grady WM (2011) Epigenetics and colorectal cancer. Nat Rev Gastroenterol Hepatol. 10.1038/nrgastro.2011.173 PMC3391545 22009203

[3] Powell SM, Zilz N, Beazer-Barclay Y, Bryan TM, Hamilton SR, et al (1992) APC mutations occur early during colorectal tumorigenesis. Nature 359: 235–237. 1528264 10.1038/359235a0

[4] Kinzler KW, Vogelstein B (1996) Lessons from hereditary colorectal cancer. Cell 87: 159–170. 8861899 10.1016/s0092-8674(00)81333-1

[5] Fang DC, Luo YH, Yang SM, Li XA, Ling XL, et al (2002) Mutation analysis of APC gene in gastric cancer with microsatellite instability. World J Gastroenterol 8: 787–791. 12378616 10.3748/wjg.v8.i5.787 PMC4656562

[6] Chai H, Brown RE (2009) Field effect in cancer-an update. Ann Clin Lab Sci 39: 331–337. 19880759

[7] Venkatachalam R, Ligtenberg MJ, Hoogerbrugge N, de Bruijn DR, Kuiper RP, et al (2010) The epigenetics of (hereditary) colorectal cancer. Cancer Genet Cytogenet 203: 1–6. 20951312 10.1016/j.cancergencyto.2010.08.013

[8] Shen L, Kondo Y, Rosner GL, Xiao L, Hernandez NS, et al (2005) MGMT promoter methylation and field defect in sporadic colorectal cancer. J Natl Cancer Inst 97: 1330–1338. 16174854 10.1093/jnci/dji275

[9] Nosho K, Kure S, Irahara N, Shima K, Baba Y, et al.. (2009) A prospective cohort study shows unique epigenetic, genetic, and prognostic features of synchronous colorectal cancers. Gastroenterology 137: 1609–1620 e1601–1603. 10.1053/j.gastro.2009.08.002 PMC2859181 19686742

[10] Braakhuis BJ, Tabor MP, Kummer JA, Leemans CR, Brakenhoff RH (2003) A genetic explanation of Slaughter's concept of field cancerization: evidence and clinical implications. Cancer Research 63: 1727–1730. 12702551

[11] Backman V, Roy HK (2011) Light-scattering technologies for field carcinogenesis detection: a modality for endoscopic prescreening. Gastroenterology 140: 35–41. 21078318 10.1053/j.gastro.2010.11.023 PMC3319699

[12] Pradhan P, Damania D, Joshi HM, Turzhitsky V, Subramanian H, et al (2011) Quantification of nanoscale density fluctuations using electron microscopy: Light-localization properties of biological cells. Appl Phys Lett 97: 243704. 10.1063/1.3524523 PMC3017571 21221251

[13] Subramanian H, Pradhan P, Liu Y, Capoglu IR, Rogers JD, et al (2009) Partial-wave microscopic spectroscopy detects subwavelength refractive index fluctuations: an application to cancer diagnosis. Opt Lett 34: 518–520. 19373360 10.1364/ol.34.000518 PMC2701738

[14] Subramanian H, Roy HK, Pradhan P, Goldberg MJ, Muldoon J, et al (2009) Nanoscale cellular changes in field carcinogenesis detected by partial wave spectroscopy. Cancer Res 69: 5357–5363. 19549915 10.1158/0008-5472.CAN-08-3895 PMC2802178

[15] Marks PA, Richon VM, Breslow R, Rifkind RA (2001) Histone deacetylase inhibitors as new cancer drugs. Curr Opin Oncol 13: 477–483. 11673688 10.1097/00001622-200111000-00010

[16] Minucci S, Pelicci PG (2006) Histone deacetylase inhibitors and the promise of epigenetic (and more) treatments for cancer. Nat Rev Cancer 6: 38–51. 16397526 10.1038/nrc1779

[17] Kawai H, Li H, Avraham S, Jiang S, Avraham HK (2003) Overexpression of histone deacetylase HDAC1 modulates breast cancer progression by negative regulation of estrogen receptor alpha. Int J Cancer 107: 353–358. 14506733 10.1002/ijc.11403

[18] Ashktorab H, Belgrave K, Hosseinkhah F, Brim H, Nouraie M, et al (2009) Global histone H4 acetylation and HDAC2 expression in colon adenoma and carcinoma. Dig Dis Sci 54: 2109–2117. 19057998 10.1007/s10620-008-0601-7 PMC2737733

[19] Nakagawa M, Oda Y, Eguchi T, Aishima S, Yao T, et al (2007) Expression profile of class I histone deacetylases in human cancer tissues. Oncol Rep 18: 769–774. 17786334

[20] Suzuki J, Chen YY, Scott GK, Devries S, Chin K, et al (2009) Protein acetylation and histone deacetylase expression associated with malignant breast cancer progression. Clin Cancer Res 15: 3163–3171. 19383825 10.1158/1078-0432.CCR-08-2319 PMC3746548

[21] Zhu P, Martin E, Mengwasser J, Schlag P, Janssen KP, et al (2004) Induction of HDAC2 expression upon loss of APC in colorectal tumorigenesis. Cancer Cell 5: 455–463. 15144953 10.1016/s1535-6108(04)00114-x

[22] Marks PA, Richon VM, Rifkind RA (2000) Histone deacetylase inhibitors: inducers of differentiation or apoptosis of transformed cells. J Natl Cancer Inst 92: 1210–1216. 10922406 10.1093/jnci/92.15.1210

[23] Ropero S, Esteller M (2007) The role of histone deacetylases (HDACs) in human cancer. Mol Oncol 1: 19–25. 19383284 10.1016/j.molonc.2007.01.001 PMC5543853

[24] Taddei A, Roche D, Bickmore WA, Almouzni G (2005) The effects of histone deacetylase inhibitors on heterochromatin: implications for anticancer therapy? EMBO Rep 6: 520–524. 15940285 10.1038/sj.embor.7400441 PMC1369099

[25] Livak KJ, Schmittgen TD (2001) Analysis of relative gene expression data using real-time quantitative PCR and the 2(−Delta Delta C(T)) Method. Methods 25: 402–408. 11846609 10.1006/meth.2001.1262

[26] Kunte DP, Wali RK, Koetsier JL, Hart J, Kostjukova MN, et al (2005) Down-regulation of the tumor suppressor gene C-terminal Src kinase: an early event during premalignant colonic epithelial hyperproliferation. FEBS Lett 579: 3497–3502. 15961079 10.1016/j.febslet.2005.05.030

[27] Damania D, Subramanian H, Tiwari AK, Stypula Y, Kunte D, et al (2010) Role of cytoskeleton in controlling the disorder strength of cellular nanoscale architecture. Biophys J 99: 989–996. 20682278 10.1016/j.bpj.2010.05.023 PMC2913198

[28] Kunte DP, Wali RK, Koetsier JL, Roy HK (2008) Antiproliferative effect of sulindac in colonic neoplasia prevention: role of COOH-terminal Src kinase. Mol Cancer Ther 7: 1797–1806. 18644992 10.1158/1535-7163.MCT-08-0022 PMC2493571

[29] Subramanian H, Pradhan P, Liu Y, Capoglu IR, Li X, et al (2008) Optical methodology for detecting histologically unapparent nanoscale consequences of genetic alterations in biological cells. Proc Natl Acad Sci U S A 105: 20118–20123. 19073935 10.1073/pnas.0804723105 PMC2629261

[30] Kramer OH, Zhu P, Ostendorff HP, Golebiewski M, Tiefenbach J, et al (2003) The histone deacetylase inhibitor valproic acid selectively induces proteasomal degradation of HDAC2. EMBO J 22: 3411–3420. 12840003 10.1093/emboj/cdg315 PMC165640

[31] Barer R, Tkaczyk S (1954) Refractive index of concentrated protein solutions. Nature 173: 821–822. 13165653 10.1038/173821b0

[32] Davies HG, Wilkins MHF, Chayen J, Lacour LF (1954) The Use of the Interference Microscope to Determine Dry Mass in Living Cells and as a Quantitative Cytochemical Method. Quarterly Journal of Microscopical Science 95: 271-&.

[33] Backman V, Kim JS, Pradhan P, Szleifer I (2011) The influence of chromosome density variations on the increase in nuclear disorder strength in carcinogenesis. Physical Biology 8. 10.1088/1478-3975/8/1/015004 21301058

[34] Zaret K (2005) Micrococcal nuclease analysis of chromatin structure. Curr Protoc Mol Biol Chapter 21: Unit 21 21. 10.1002/0471142727.mb2101s69 18265356

[35] Kramer OH, Gottlicher M, Heinzel T (2001) Histone deacetylase as a therapeutic target. Trends Endocrinol Metab 12: 294–300. 11504668 10.1016/s1043-2760(01)00438-6

[36] Wali RK, Roy HK, Kim YL, Liu Y, Koetsier JL, et al (2005) Increased microvascular blood content is an early event in colon carcinogenesis. Gut 54: 654–660. 15831911 10.1136/gut.2004.056010 PMC1262671

[37] Banerjee A, Quirke P (1998) Experimental models of colorectal cancer. Dis Colon Rectum 41: 490–505. 9559635 10.1007/BF02235764

[38] Shogren-Knaak M, Ishii H, Sun JM, Pazin MJ, Davie JR, et al (2006) Histone H4-K16 acetylation controls chromatin structure and protein interactions. Science 311: 844–847. 16469925 10.1126/science.1124000

[39] Gottlicher M, Minucci S, Zhu P, Kramer OH, Schimpf A, et al (2001) Valproic acid defines a novel class of HDAC inhibitors inducing differentiation of transformed cells. EMBO J 20: 6969–6978. 11742974 10.1093/emboj/20.24.6969 PMC125788

[40] Insinga A, Monestiroli S, Ronzoni S, Gelmetti V, Marchesi F, et al (2005) Inhibitors of histone deacetylases induce tumor-selective apoptosis through activation of the death receptor pathway. Nat Med 11: 71–76. 15619634 10.1038/nm1160

[41] Gorisch SM, Wachsmuth M, Toth KF, Lichter P, Rippe K (2005) Histone acetylation increases chromatin accessibility. J Cell Sci 118: 5825–5834. 16317046 10.1242/jcs.02689

[42] Van Lint C, Emiliani S, Verdin E (1996) The expression of a small fraction of cellular genes is changed in response to histone hyperacetylation. Gene Expr 5: 245–253. 8723390 PMC6138027

[43] Kim JS, Pradhan P, Backman V, Szleifer I (2011) The influence of chromosome density variations on the increase in nuclear disorder strength in carcinogenesis. Phys Biol 8: 015004. 21301058 10.1088/1478-3975/8/1/015004

[44] Egger G, Liang G, Aparicio A, Jones PA (2004) Epigenetics in human disease and prospects for epigenetic therapy. Nature 429: 457–463. 15164071 10.1038/nature02625

[45] Misteli T (2010) Higher-order genome organization in human disease. Cold Spring Harb Perspect Biol 2: a000794. 20591991 10.1101/cshperspect.a000794 PMC2908770

[46] Orr HT, Zoghbi HY (2007) Trinucleotide repeat disorders. Annu Rev Neurosci 30: 575–621. 17417937 10.1146/annurev.neuro.29.051605.113042

[47] Alberts DS, Einspahr JG, Krouse RS, Prasad A, Ranger-Moore J, et al (2007) Karyometry of the colonic mucosa. Cancer Epidemiol Biomarkers Prev 16: 2704–2716. 18086777 10.1158/1055-9965.EPI-07-0595

[48] Richon VM, Sandhoff TW, Rifkind RA, Marks PA (2000) Histone deacetylase inhibitor selectively induces p21WAF1 expression and gene-associated histone acetylation. Proc Natl Acad Sci U S A 97: 10014–10019. 10954755 10.1073/pnas.180316197 PMC27656

[49] Zhao Y, Tan J, Zhuang L, Jiang X, Liu ET, et al (2005) Inhibitors of histone deacetylases target the Rb-E2F1 pathway for apoptosis induction through activation of proapoptotic protein Bim. Proc Natl Acad Sci U S A 102: 16090–16095. 16243973 10.1073/pnas.0505585102 PMC1276064

[50] Roy HK, Subramanian H, Damania D, Hensing TA, Rom WN, et al (2010) Optical detection of buccal epithelial nanoarchitectural alterations in patients harboring lung cancer: implications for screening. Cancer Res 70: 7748–7754. 20924114 10.1158/0008-5472.CAN-10-1686 PMC3703950

[51] Michor F, Liphardt J, Ferrari M, Widom J (2011) What does physics have to do with cancer? Nat Rev Cancer 11: 657–670. 21850037 10.1038/nrc3092 PMC3711102

